# Does compulsion explain addiction?

**DOI:** 10.1111/adb.13379

**Published:** 2024-04-08

**Authors:** Andreas Heinz, Stefan Gutwinski, Nadja Samia Bahr, Rainer Spanagel, Gaetano Di Chiara

**Affiliations:** ^1^ Department of Psychiatry and Neuroscience|CCM, NeuroCure Clinical Research Center, Berlin Institute of Health CCM, Charité‐Universitätsmedizin Berlin, Freie Universität Berlin Humboldt‐Universität zu Berlin Berlin Germany; ^2^ German Center for Mental Health (DZPG) Berlin‐Potsdam; ^3^ Institute for Psychopharmacology, Medical Faculty Mannheim, Central Institute of Mental Health (CIMH) Heidelberg University Mannheim Germany; ^4^ Department of Biomedical Sciences, University of Cagliari Cittadella Universitaria di Monserrato Cagliari Italy; ^5^ Neuroscience Institute National Research Council of Italy (CNR) Cagliari Italy

**Keywords:** addiction, animals, compulsive behaviour, drug‐seeking behaviour, habits

## Abstract

One of the leading drug addiction theories states that habits and the underlying neural process of a ventral to dorsal striatal shift are the building blocks of compulsive drug‐seeking behaviour and that compulsion is the maladaptive persistence of responding despite adverse consequences. Here we discuss that compulsive behaviour as defined primarily from the perspective of animal experimentation falls short of the clinical phenomena and their neurobiological correlates. Thus for the human condition, the concept of compulsive habbits should be critically addressed and potentially revised.

## 1 INTRODUCTION

Compulsion is often regarded as a key feature of addictions and obsessive‐compulsive disorders (OCD).^1^ One of the leading drug addiction theories describes addictive behaviour as a transition from voluntary goal‐directed, recreational drug use to compulsive drug‐seeking habits that result from prefrontal cortical to striatal control over drug‐seeking as well as a progression from the ventral to the dorsal striatum.^2^, ^3^ Thus, an individual may compulsively seek drugs – that is, persist in seeking drugs despite the negative consequences of doing so – when executive control over this maladaptive behaviour is diminished and when the neural systems that underlie habitual behaviour dominate goal‐directed behavioural systems.^4^ In other words, habits and the ventral to dorsal striatal shift are the building blocks of compulsive drug‐seeking behaviour.

Here, we argue that this theory may adequately characterize drug‐seeking behaviour in certain animal studies but does not adequately describe human addictive behaviour. In particular, we argue that, in humans, obsessive and compulsive behaviours, as observed in the OCD spectrum, differ significantly from addictive behaviour, and that a shift from goal‐directed towards habitual behaviour does not necessarily lead to compulsive behaviour.

## 2 HUMAN OBSESSIVE‐COMPULSIVE DISORDERS (OCD) DIFFER SIGNIFICANTLY FROM ADDICTIONS

Although symptoms of OCD have been compared with obsessive craving and ruminating about drug consumption as well as compulsive drug taking in spite of adverse consequences, compulsive behaviour in OCD has a different meaning than in addictions. In OCD, obsessive thoughts usually trigger compulsive behaviour which in turn helps to dispel the thoughts and reduce distress, whereas in addictions compulsive behaviour is defined as the maladaptive persistence of responding despite adverse consequences.

The first indication that obsessions and compulsions in OCD differ substantially from addictive behaviour is based on clinical observation. Thus, individuals with both OCD and addiction disorders can clearly distinguish between such symptoms and are able to describe substantial differences in their respective experiences. For example, a patient suffering from OCD, alcohol dependence and pathological gambling described that with OCD, he always had to perform certain rituals in order to cope with his aversive obsessions. Interruption of ritualistic behaviour or postponement of its initiation was so highly aversive that it was only possible for short moments of time. His experience was quite different with respect to alcohol craving, which was elicited unexpectedly by alcohol‐associated cues, and differed from his craving for gambling, which usually occurred at 6 p.m., when he was previously able to engage in online gambling.^5^ Furthermore, obsessions usually focus on aversive thought content, for example being somewhat *impure* or *soiled*, or on highly aversive, aggressive thoughts directed against other human beings including children.^6^ Ritualistic behaviour often aims at compensating for the negative, *evil* thoughts, which are experienced as inadequate and highly threatening by the afflicted individual.^6^, ^7^ Craving for alcohol or other drugs of abuse, on the other hand, represents craving for a certain reward, which is at least in the initial stages of drug dependence associated with a positive mood state^8^ but at a more severe stage, craving can be also induced by a negative state such as conditioned drug withdrawal or stress.^9^

## 3 NEURAL CORRELATES OF OCD DIFFER SIGNIFICANTLY FROM ADDICTIONS

A second substantial difference between OCD and addictive behaviour is in their neurobiological correlates: in the frontal cortex, which has a key role in goal‐directed behaviour, increased glucose utilization was found in patients with OCD.^10^, ^11^ Accordingly, successful treatment with antidepressants or behaviour therapy was associated with a significant decrease in glucose utilization in the orbitofrontal cortex.^10^, ^11^, ^12^ This is in contrast to neurobiological correlates of individuals with drug addiction: glucose utilization in the frontal cortex was repeatedly reported to be reduced.^13^, ^14^

For example, Volkow and co‐workers^13^ found a reduction of whole brain glucose metabolism during rest in heavy drinkers, and Adams and colleagues^15^ an association between low glucose utilization in the medial prefrontal cortex and increased errors in the Wisconsin Card Sorting Test that assesses executive functions. Furthermore, alterations in dopamine neurotransmission as evinced by a reduction of dopamine D2 receptor availability in the striatum were associated with low levels of orbitofrontal glucose utilization in individuals with methamphetamine abuse.^14^ Finally, in addictive behaviours, low availability of dopamine D2 receptors following immediate detoxification has repeatedly been described in the striatum,^16^, ^17^ while in the OCD spectrum, increased D2 receptor availability was associated with motor tic manifestation.^18^ Finally, in OCD, more complex obsessive and compulsive symptoms were associated with serotonergic alterations in the brainstem,^19^ an alteration that is not regularly seen in individuals with addictive behaviour.^20^

Despite these dissimilarities between OCD and addictions on the behavioural and neural level, some studies suggest that there are similarities between the obsessive‐compulsive spectrum and addictive behaviour, particularly a general reduction in goal‐directed behaviour and a shift towards habitual behaviour.^2^, ^3^ However, a reduction in goal‐directed behaviour may occur due to different neural processes: it may e.g. result from the neurotoxic effects of alcohol or other drugs on the frontal cortex or be related to an overactivation of frontal cortical‐striatal circuits in OCD.^10^, ^21^, ^22^ These different effects may lead to a shift towards habitual behaviour in addictions as well as in OCD. However, habits should not necessarily be considered as building blocks for compulsive behaviour unless they occur in spite of immediate aversive consequences, which is usually not the case in addiction. This will be discussed in the following.

## 4 A SHIFT FROM GOAL‐DIRECTED TOWARDS HABITUAL BEHAVIOUR DOES NOT NECESSARILY LEAD TO COMPULSIVE BEHAVIOUR

A significant contribution of habitual behaviour in human drug addiction^6^, ^23^, ^24^ is disputed by the group of Lee Hogarth, who showed that, in some but not all studies, goal‐directed behaviour is preserved, as e.g. indicated by intact effects of the devaluation of outcomes among human persons with drug addiction.^25^, ^26^ Furthermore, Hogarth argues that effects of drug cues do not correlate with addiction severity, as shown e.g. in the lack of significant differences in drug cue effects between addicted and non‐addicted persons.^27^ Hogarth's approach refers to the so‐called dual systems theory, which suggests that habitual decision‐making relies on a neural system that is completely independent of the brain system underlying goal‐directed decision.^10^, ^22^ However, there is a relevant overlap in the neural correlates of brain areas that underlie goal‐directed and habitual decision as shown by anatomical and functional studies in humans and non‐human primates,^28^ arguing against a strict dichotomy between these brain networks and their behavioural correlates. Furthermore, the dual systems approach does not adequately reflect complex differences in ventral striatal shell and core and their respective effects on stimulus‐drug associations on the one hand and sensitization towards drug effects on the other as evidenced in animal experiments.^29^ Regarding effects of drug‐related stimuli in humans, we and others observed significant associations between Pavlovian conditioned non‐drug and drug cues on unrelated instrumental behaviour in so‐called Pavlovian‐to‐Instrumental Transfer tasks as well as cue‐induced brain activation^30^, ^31^ on the one hand, and addiction severity as indicated by the prospective relapse risk on the other.^31^, ^32^, ^33^ Confirming the relevance of human abilities to reflect on ongoing behaviour, drug cues tended to promote behavioural withdrawal (and not approach) in patients with alcohol dependence who managed to abstain after detoxification, while this was not the case in subsequent relapsers or controls.^34^

Altogether, we suggest that habit formation plays a significant role in drug addiction, as long as habitual behaviour is regarded as dimensionally but not categorically different from goal‐directed decision‐making and modifiable by human cognition. It was indeed shown that an increase in habitual behaviour is associated with the severity of drug use, in this case, relapse after detoxification, but only if it is accompanied by high alcohol expectations.^35^ These findings suggest that Pavlovian cues and a shift from goal‐directed towards habitual behaviour may contribute to automatic drug intake as suggested by Tiffany and Carter.^36^ However, humans appear to be able to reflect upon their behaviour and are usually able to change it, also evidenced by the 10 to 20% of alcohol‐dependent patients who manage to *kick the habit* without professional help by achieving abstinence or extensively reducing drug intake.^37^, ^38^, ^39^, ^40^

This is certainly not the case in animal experiments where animals are defined as habitual by reward devaluation testing; they are simply not able to *kick the habit* by intrinsic motivation. But do laboratory animals such as mice or rats that show a specific task behaviour show also compulsive behaviour? This is usually tested by punishment; i.e., if an animal goes for a conditioned reward (in this case the drug cue) despite the fact that it receives a foot‐shock it is considered as compulsive. However, drug taking in spite of aversive consequences can also occur without previous habit formation in rodents.^41^ Moreover, does this definition of compulsion as maladaptive persistence of responding despite adverse consequences also hold true for the human condition?

We argue that this is not the case in human addiction. In fact, aversive outcomes of repeated drug intake often take months or years to manifest. This is not only true for somatic illnesses, such as liver cirrhosis, neurotoxicity or cancer following long‐term alcohol intake,^42^, ^43^ but also for negative consequences in the form of social problems associated with repeated drug intake, even more so because such consequences vary widely depending on whether a certain drug can be legally consumed, as is the case in certain countries.^44^ In the case of illegal drug consumption, immediate negative consequences may occur, for example when an individual gets arrested while trying to acquire an illegal drug. However, such socially variant conditions depend on national legislation and are not characteristics of the individual with an addictive behaviour.

Since the legality of a drug differs widely not only between nations but also in the history of societies (alcohol prohibition in the United States of America being a prominent example), the sociocultural framework of drug intake is hardly comparable across and within different countries.^45^ Accordingly, the replacement of the well‐defined concept of dependence^46^ by a dimensional approach, as defined in the DSM‐5, which includes social problems, may lead to inconsistent classification, as some sociocultural settings may only occur in some countries.^47^, ^48^, ^49^

In summary, even if an individual is severely dependent negative consequences including life‐threatening somatic diseases may usually not occur immediately following repeated drug intake.^43^ This punishment discounting, which is scarcely studied in humans, differs substantially from immediate punishment in animal experiments focusing mainly on drug‐seeking seeking behaviour.^4^ Additionally, in most animal experiments animals are in isolation, which is unusual for rodents and may influence motivation for drug taking.^50^, ^51^ In some animal experiments, environmental enrichment reduced the acquisition of drug seeking and facilitated drug abstinence.^52^, ^53^ Animal experiments thus often differ substantially from human studies with respect to social settings as well as very basic settings such as the delay between drug taking and punishment, which limits the comparability to humans. Therefore, the concept that habits and the ventral to dorsal striatal shift are the building blocks of compulsive drug‐seeking behaviour and that compulsion is the maladaptive persistence of responding despite adverse consequences in humans should be critically addressed and potentially revised.

## 5 CONCLUSIONS

Our considerations do not neglect that individuals with addictions may have habitual behaviour and that it can be very difficult to *kick the habit* of drug intake. However, we hypothesize that such habits should not be described as a compulsion that is carried out irrespective of immediate aversive consequences under all circumstances. Individuals with drug addiction are neither automatons that carry out habitual behaviour without cognitive control nor are they just individuals with bad choices. Repeated drug consumption has profound impacts on motivationally and cognitively relevant neurocircuits and may bias an individual towards repeated drug intake.^6^ Compared to other human behaviours, such as trained movements in sports, it can be extremely difficult to change habitual behaviour. This is particularly true when drug addiction is accompanied by a lack of alternative rewards that are at least in the long run healthier for the individual. Accordingly, social deprivation and poverty, which may limit access to alternative rewards, should regularly be considered in human studies. Altogether, we feel that compulsive behaviour as defined primarily from the perspective of animal experimentation falls short of the clinical phenomena and their neurobiological correlates.

## Data Availability

Data sharing not applicable to this article as no datasets were generated or analysed during the current study.
